# Optical shaping of the polarization anisotropy in a laterally coupled quantum dot dimer

**DOI:** 10.1038/s41377-020-0339-3

**Published:** 2020-06-11

**Authors:** Heedae Kim, Kwangseuk Kyhm, Robert A. Taylor, Jong Su Kim, Jin Dong Song, Sungkyun Park

**Affiliations:** 1 School of Physics, Northeast Normal University, 130024 Changchun, China; 2 Clarendon Laboratory, Department of Physics, University of Oxford, Oxford, OX1 3PU UK; 3 Department of Opto-mechatronics, Pusan Nat’l University, Busan, 609-735 Republic of Korea; 4 Department of Physics, Yeungnam University, Gyeongsan, 712-749 Republic of Korea; 5 Nano-Photonics Research Center, KIST, Seoul, 136-791 Republic of Korea; 6 Department of Physics, Pusan Nat’l University, Busan, 609-735 Republic of Korea

**Keywords:** Micro-optics, Single photons and quantum effects

## Abstract

We find that the emission from laterally coupled quantum dots is strongly polarized along the coupled direction [1$\bar 1$0], and its polarization anisotropy can be shaped by changing the orientation of the polarized excitation. When the nonresonant excitation is linearly polarized perpendicular to the coupled direction [110], excitons (X_1_ and X_2_) and local biexcitons (X_1_X_1_ and X_2_X_2_) from the two separate quantum dots (QD_1_ and QD_2_) show emission anisotropy with a small degree of polarization (10%). On the other hand, when the excitation polarization is parallel to the coupled direction [1$\bar 1$0], the polarization anisotropy of excitons, local biexcitons, and coupled biexcitons (X_1_X_2_) is enhanced with a degree of polarization of 74%. We also observed a consistent anisotropy in the time-resolved photoluminescence. The decay rate of the polarized photoluminescence intensity along the coupled direction is relatively high, but the anisotropic decay rate can be modified by changing the orientation of the polarized excitation. An energy difference is also observed between the polarized emission spectra parallel and perpendicular to the coupled direction, and it increases by up to three times by changing the excitation polarization orientation from [110] to [1$\bar 1$0]. These results suggest that the dipole–dipole interaction across the two separate quantum dots is mediated and that the anisotropic wavefunctions of the excitons and biexcitons are shaped by the excitation polarization.

## Quantum dots: coupled control

New information storage technologies could use polarized light to control the optical properties of coupled quantum dots. The idea of combining quantum dots – nanoscale semiconductor crystals – into coupled pairs has attracted great attention due to the increased number of exotic quantum states that can be realized for storing data. Robert Taylor at the University of Oxford, UK, with co-workers in China and Korea, directed polarized laser light onto coupled gallium arsenide quantum dots to induce photoluminescence. They found that the emitted light showed very different characteristics depending on whether the excitation was parallel or at right angles to the line formed by the two dots. This ‘optical shaping’ reflects different arrangements of excitons – bound states of electrons and holes – in the coupled dots, and could open new avenues for data storage and thermoelectric energy harvesting.

## Introduction

Coupled quantum dots (CQDs) are considered an important building block in the development of scalable quantum devices by controlling the coupled states of two adjacent quantum dots (QDs) electrically and optically^1–9^, and this approach can be further extended in the context of cavity quantum electrodynamics. For example, a long-lived two-spin-cavity system can be obtained when a pair of QDs are embedded in a photonic crystal cavity^10^. Currently, CQDs are generating great interest as an emerging topic in phononics. Provided that a molecular polaron is formed in a CQD, the phonon-induced transparency results in Fano-type quantum interference^11^. CQDs can also be used in thermoelectric energy harvesting. When two QDs are coupled capacitively, the charge and heat flow directions can be decoupled^12^.

Remarkable progress has been made in controlling the coupled states of CQDs^13–25^, but individual control of vertically stacked QDs is still challenging. Unless each dot can provide a logical bit operation, this issue becomes a limiting factor in achieving scalable qubit arrays. Laterally coupled QDs can be an alternative system if the charge state and the lateral coupling of two dots (QD_1_ and QD_2_) are controlled separately. For example, tuneable vector electric fields with arbitrary magnitudes and angles can be generated in laterally coupled QDs when four electrodes are implemented in a mesa structure^15^. Recently, the droplet epitaxy method^13^ enabled the growth of laterally coupled QDs with precise morphology control. However, the center-to-center distance between two dots (QD_1_ and QD_2_) was large (R_12_ ~ 30 nm) compared with the few nm separation of vertically stacked QDs. In this case, the tunneling coupling is significantly weaker in laterally coupled QDs, while wavefunction overlap is likely to be induced in vertically coupled QDs when applying an external electric field. Therefore, coupling between laterally separate dots requires delicate control when an external field is applied.

Although tunneling-induced coupling is very unlikely with the large dot-to-dot distance of a laterally coupled QD structure, the separate excitons in the two QDs can be coupled through the dipole–dipole interaction^26,27^. Specifically, two kinds of mechanisms, Forster energy transfer (FRET) and the direct Coulomb interaction, are involved in the exciton dipole–dipole interaction, although both mechanisms have the same ~$1/R_{12}^3$ dependence with the interdot distance (~R_12_). While the transition dipole moment determines the strength of FRET, the permanent dipole moment governs the direct Coulomb interaction, where the permanent dipole moment of an exciton originates from a shift of the electron and hole charge distributions. Both mechanisms depend on the spatial arrangement of dipoles, such as the orientation and charge distribution. Recently, we found that the FRET interaction becomes dominant compared with the direct Coulomb interaction when the excitation polarization is parallel to the lateral coupled direction^27^. As a result, the photoluminescence (PL) spectra of excitons and biexcitons shows a spectral redshift as well as population transfer when the polarized excitation along the coupled direction is increased up to 25 Wcm^−2^. However, neither a redshift nor population transfer is observed when the excitation polarization is perpendicular to the coupled direction. Therefore, the coupling of laterally coupled QDs can be controlled by the excitation polarization. In this work, the anisotropic wavefunctions of laterally coupled QDs controlled by the excitation polarization are revealed in terms of the anisotropy of the emission intensity and decay rate. In particular, a limited excitation intensity (7 Wcm^−2^) was used. In this case, exciton population transfer is not activated even with linear polarization along the coupled direction. However, the alignment orientation dependence of the exciton dipole–dipole interaction is still valid, although the two dots are laterally separated with a center-to-center distance of ~30 nm. Consequently, we found that optical shaping of the polarization anisotropy in laterally coupled QDs is possible, where the spatial arrangement of excitons and biexcitons can be controlled by the excitation polarization.

## Results and discussion

**Fig. 1:**
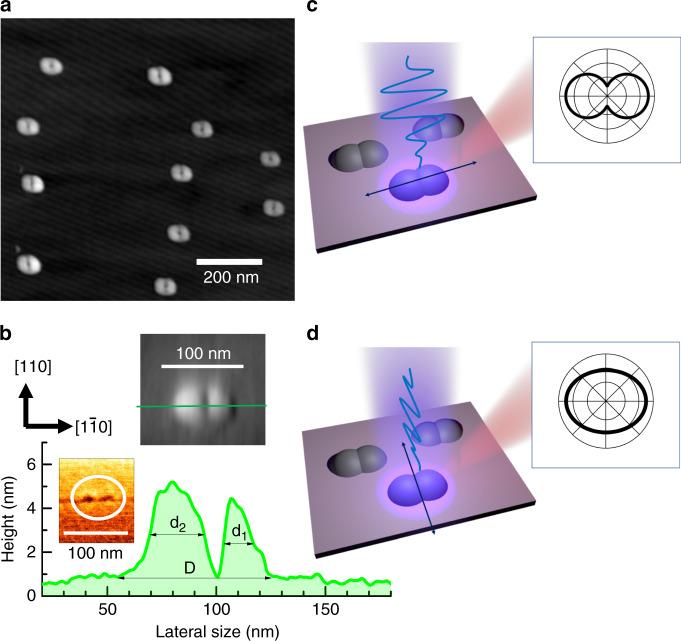
Schematic showing atomic force microscopy (AFM) images and cross-sections of uncapped GaAs CQDs. (**a**) In a top view, where the height profile of a selected single CQD is shown along the coupled direction (**b**). Note that the two dots (QD_1_ and QD_2_) are separated with a center-to-center distance of ~30 nm, and the inset shows a cross-sectional scanning electron microscopy (SEM) image after AlGaAs barrier capping. For excitation polarized parallel (**c**) (*θ*_ex_ = 90°) and perpendicular (**d**) (*θ*_ex_ = 0°) to the coupled direction [1$\bar 1$0], different anisotropies are obtained in the photoluminescence intensity

Figure 1a shows an Atomic force microscopy (AFM) image of uncapped GaAs CQDs in a top view, where droplet dots are paired along the coupled direction [1$\bar 1$0]. Because the spatial resolution of an AFM measurement is often limited by tip convolution effects due to the curvature of the tip, we performed AFM measurements in the noncontact mode (NC-AFM) with a carbon nanotube, resulting in an enhancement of the resolution. According to this technique, we measured the average size of CQDs along [1$\bar 1$0] (~60 nm) and [110] (~40 nm), and the QDs have a 10% size inhomogeneity. In Fig. 1b, the height profile of a single CQD structure is shown along [1$\bar 1$0], and the baseline (*D* = 62.0 nm) and full width at half maximum dot heights (*d*_1_ = 23 nm and *d*_2_ = 12 nm) were measured for the two different dots. It should be noted that the two dots of a laterally coupled QD dimer are separated with a center-to-center distance of ~30 nm, while tunneling-based CQDs are separated with an interdot distance of several nanometers^1,16,18–20,24^. In the case where a tunneling effect is observed between two adjacent QDs, indirect excitons need to be considered, where the electrons and the holes belong to different QDs. With increasing external electric field, the indirect and direct excitons can be in resonance. As a result, the bonding and antibonding states of a QD dimer appear. Nevertheless, the emission intensity of the indirect excitons is weak due to the small overlap of the electron and hole wavefunctions unless the external electric field is sufficiently strong. Because the height of the QDs is smaller than the lateral dimensions by an order of magnitude, the lateral potential valleys of electrons (*V*_e_) and heavy holes (*V*_hh_) can be obtained from the height morphology (Supplementary Information), whereby we found that the ground states of electrons and holes are separated by a thick potential barrier (~12 nm). Therefore, the two separate excitons are likely coupled only through the dipole–dipole interaction instead of through tunneling.

The dipole–dipole interaction depends on the relative dipole orientations, and the orientation of an exciton is determined by the spatial distribution of electrons and holes. Provided that the distribution of electrons and holes is determined by the excitation polarization, the dipole–dipole interaction can be controlled optically. As shown schematically in Fig. 1c, nonresonant excitation polarized along [1$\bar 1$0] gives rise to a significant anisotropy in the photoluminescence of excitons. On the other hand, with excitation polarized perpendicular to the coupled direction ([1$\bar 1$0]), a different PL anisotropy is obtained, as shown in Fig. 1d. Currently, the exact reasons have not been clarified, but three aspects need to be considered to find the origin. First, when carriers are excited by polarized light, the polarization of the excitation light is transferred to the spin of excited electron–hole pairs. With polarized nonresonant excitation, spin-polarized carriers are injected from the surrounding barrier into the QDs, and the initial spin coherence likely deteriorates during intra-relaxation through various elastic and inelastic scatterings with phonons. Because the exciton states are vulnerable to spin flip due to the electron–hole exchange interaction, it is known that the spins of uncorrelated separate electrons and holes are better conserved during spin injection than the spins of correlated electrons and holes in an exciton, and the same tendency was observed in different structures such as in QDs, laterally coupled QDs, and quantum rings^28^. Second, nonresonantly excited carriers are also known to affect the electric dipole of excitons via coherent many-body interactions in various quantum confinement structures^29–31^. For example, the confinement states of excitons and the continuum states can be combined as a single coherent state instead of independent transitions in quantum wells^29^, and nonresonant excitation of QDs affects the ground state exciton as a consequence of the ultrafast interaction with the QD environment^30^. Third, in GaAs/AlGaAs droplet quantum structures, it was found that the exciton wavefunction can be modified by increasing nonresonant excitation. If photoexcited electrons and holes are trapped at the interface defects of quantum structures, then a local electric field can be induced^31^. Provided that linearly polarized excitation gives rise to a charge distribution around quantum structures, this may explain the excitation polarization dependence. In addition, the charge states with separate electrons and holes are known to affect the photon emission efficiency of the QD ground state. In the case of pulsed nonresonant excitation, the photon emission of the QD ground state followed by the anti-bunching at zero delay time shows significant bunching. However, consecutive bunching becomes suppressed with pulsed nonresonant excitation^32^. Similarly, the charged states near QDs also give rise to spectral diffusion^33^. Because our laterally coupled GaAs/AlGaAs QD structures are also grown by the droplet method, nonresonant excitation likely induces a local electric field (*E*_loc_) via the trapped photoexcited carriers. Therefore, the dipole of the ground state exciton can be affected by the polarization of nonresonant excitation light, and optical shaping of the emission polarization and the wavefunction can be possible.

For dense Stranski–Krastanov QDs (~10^3^ μm^−2^), transmission electron microscopy (TEM) can be used to measure a cross-sectional image of the QDs instead of AFM. However, the low density (~10 μm^−2^) of droplet QDs here makes acquiring a cross-sectional TEM image very challenging^34^, as finding a quantum dot along the cleaved sample edge is very unlikely. Furthermore, the TEM image contrast between GaAs and AlGaAs would be poor due to the low Al content in the alloy. As an alternative method, we used scanning electron microscopy (SEM) to obtain a cross-sectional image of GaAs QDs embedded in an AlGaAs barrier (inset of Fig. 1b). Recently, it was found that the lattice-matched system of GaAs/AlGaAs droplet QDs results in no strain effects, where atomic scale analysis was employed using cross-sectional scanning tunneling microscopy^35^; i.e., the separation between bilayers in a GaAs QD is barely affected when GaAs droplet QDs are capped by AlGaAs. Although Al intermixing occurs, the Al concentration in GaAs QDs is not significant (~6%). Nevertheless, the atomic arrangement of AlGaAs adjacent to GaAs QDs is found to be nonuniform. An Al-rich region is formed on top of the dot due to the different mobilities of Al and Ga atoms during AlGaAs capping, and AlAs-rich regions are also formed due to Ga desorption during thermal annealing. Therefore, these nonuniform barrier regions are possibly associated with the local field induced by the trapped photoexcited carriers.

Provided that the exciton wavefunction of a QD is elliptical, two split PL spectra of excitons can be observed. Regarding the node configuration of the exciton polarization distribution and the geometric aspect ratio of an elliptical QD, fine splitting can be predicted theoretically based on the short- and long-range electron–hole exchange interaction^36,37^. As the two eigenstates are defined based on the two perpendicular symmetric axes, the linear polarizations of the split PL spectra are perpendicular to each other. In the droplet growth method (Supplementary Information), the morphology of nanostructures can be controlled by changing the amount of As flux. While large QDs are formed with high As flux (~10^−4^ Torr), quantum rings are formed with low As flux (~10^−6^ Torr). However, with an intermediate As flux (~10^−5^ Torr), a dip is formed in the central part of a large droplet quantum dot, and the rim becomes disconnected. Consequently, separate QDs are grown. In this case, the lateral shape of separate QDs is crescent-like^38^ rather than elliptical. Therefore, the typical selection rule for elliptical QDs is no longer valid due to the reduced symmetry. For example, the split singlet biexciton state (XX) in an elliptical QD corresponding to the high energy exciton (X) with the same vertical (0°) linear polarization appears at low energy, but the other polarized biexciton state paired with the low energy exciton with the same horizontal (90°) polarization appears at higher energy^37^. The cascade emission of the polarized XX–X pairs can be verified through the polarization dependence of the single photon cross-correlation when each XX and X shows anti-bunching in the autocorrelation of the Hanbury Brown and Twiss measurement. With the same collinear polarizations, the second-order correlation *g*^2^ between XX and X shows significant bunching as evidence of cascade photon emission. However, suppressed bunching is observed with the cross-linear polarization pair of XX and X. The triplet biexciton states in an elliptical QD, however, show opposite results^39^, and the novel polarization dependence originates from the *p*-orbital symmetry of the excited hole state. Therefore, the polarizations of cascade XX–X emission in our laterally coupled QDs are likely cross-linear provided that a *p*-like symmetry is induced in crescent-like QDs.

**Fig. 2:**
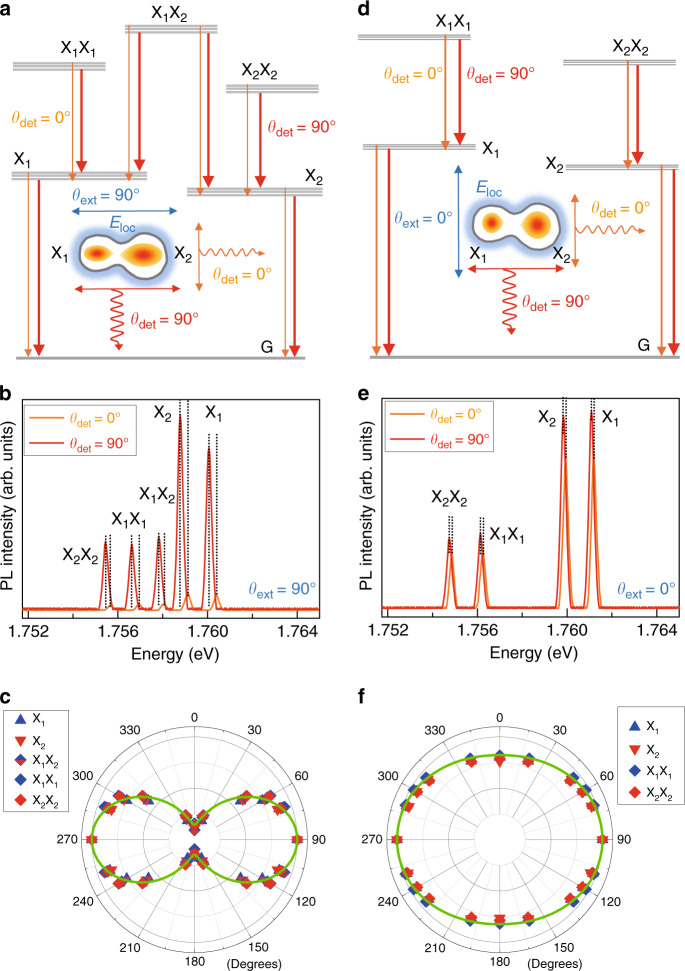
Schematic of the emission polarization dependence of the PL. With excitation polarized parallel to the coupled direction (*θ*_ext_ = 90°) (**a**), the polarized PL spectra parallel (*θ*_det_ = 90°) and perpendicular (*θ*_det_ = 0°) to the excitation direction are measured separately (**b**). **c** The PL anisotropy with detection angle (*θ*_det_) is shown in terms of the normalized intensity. With excitation polarized perpendicular to the coupled direction (*θ*_det_ = 0°) (**d**), the polarized PL spectrum at *θ*_det_ = 90° is still dominant, but the polarized PL spectrum at *θ*_det_ = 0° is enhanced (**e**). As a result, the PL anisotropy with *θ*_ext_ = 0° excitation is suppressed (**f**) compared with that with *θ*_ext_ = 90° excitation

As shown schematically in Fig. 2a, a narrow polarized spectrum can be selected by an analyzer, where lateral azimuthal angles are defined for linearly polarized detection (*θ*_det_) and excitation (*θ*_ext_). For example, when linearly polarized excitation is parallel to the coupled direction (*θ*_ext_ = 90°), the split PL spectrum can be selected with two perpendicular detection angles of *θ*_det_ = 0° and *θ*_det_ = 90°. Figure 2b shows a micro-PL spectrum of a single laterally coupled QD structure with nonresonant (~3.1 eV) excitation polarized along the coupled direction (*θ*_ext_ = 90°). The PL spectrum polarized in the same direction (*θ*_det_ = 90°) shows a redshift with a large intensity compared with that polarized in the perpendicular direction (*θ*_det_ = 0°). Regarding the confinement size dependence of the exciton oscillator strength, this result suggests that the wavefunction has a large extent along the coupled direction (*θ*_det_ = 90°) compared with that along the perpendicular direction (*θ*_det_ = 0°). In general, the wavefunctions of electrons and holes are determined by the shape of confinement structures, but the excitation polarization seems to affect the distribution of electrons and holes via *E*_loc_. Suppose that excitation polarized along *θ*_ext_ induces *E*_Loc_ via the trapped charges, and the wavefunction can also be affected by *θ*_ext_. With *θ*_ext_ = 90°, the exciton wavefunctions (X_1_ and X_2_) can be elongated along the coupled direction. As a result, the dipole oscillator strength of X_1_ and X_2_ increases^40^, and the polarized emission intensity of *θ*_det_ = 90° also increases compared with that of *θ*_det_ = 0°.

Because of the size difference between the two dots (QD_1_ and QD_2_), an energy difference (~1.3 meV) is observed in the PL spectra for the two excitons (X_1_ and X_2_). We also observed local biexcitons (X_1_X_1_ and X_2_X_2_) with a binding energy of ~3.4 meV, where the biexciton nature was also confirmed by the quadratic dependence of the integrated PL intensity with the excitation intensity. As shown in Fig. 2b, both the local biexcitons and the excitons at *θ*_det_ = 90° appear at low energy compared with those at *θ*_det_ = 0°. In addition, we found that the cascade emission between local biexcitons and excitons is cross-linearly polarized in the separate QDs through the polarization dependence of the photon cross-correlation, as mentioned before. This result is in contrast to the collinear polarization pair of cascade XX–X emission in elliptical QDs. Although the novel polarization selection rule of optical transitions is not completely understood, the wavefunction asymmetry in crescent-like QDs is a possible origin.

In addition, the increased wavefunction extent along the coupled direction is also advantageous when the alignment orientation dependence of the exciton dipole–dipole interaction is considered. As the excitation is increased, the enhanced FRET dipole–dipole interaction between X_1_ and X_2_ results in a redshift with population transfer. From an energy point of view, this is a case of an attractive interaction. Therefore, a coupled biexciton (X_1_X_2_) is likely formed. Similarly, hetero-biexcitons (X_A_X_B_) were also observed in bulk GaN through four-wave mixing (FWM) spectroscopy, where the homo-biexcitons of X_A_X_A_ and X_B_X_B_ correspond to the A and B valence bands, respectively. Both the homo- and hetero-biexcitons have antibonding excited states, and the excited states are known to result in phase modulation of quantum beat FWM signals^41^. Therefore, the presence of an antibonding excited X_1_X_2_ state can also be verified through the same technique. As shown schematically in Fig. 2a, two separate transitions (X_1_X_2_–X_1_ and X_1_X_2_–X_2_) are possible from X_1_X_2_. The additional PL peak near 1.758 eV in Fig. 2b can be attributed to the X_1_X_2_–X_1_ transition. When the two X_1_ generated by *θ*_ext_ = 90° excitation are paired in the same QD_1_, a binding energy of ~3.4 meV is seen for the local biexciton (X_1_X_1_). Alternatively, X_1_ can also be bound to X_2_ across separate QDs with a relatively small binding energy of ~2.2 meV, which corresponds to the energy difference between X_1_ and X_1_X_2_. For *θ*_ext_ = 90° excitation (Fig. 2(b)), it is notable that the PL intensity of X_2_ is large compared with that of X_1_. On the other hand, for *θ*_ext_ = 0° excitation (Fig. 2e), the PL intensities of X_1_ and X_2_ are nearly balanced, and a coupled biexciton (X_1_X_2_) is not generated. These results suggest that the X_1_X_2_–X_2_ transition possibly overlaps with the PL linewidth of X_2_. In a preliminary experiment^27^, this conjecture was verified in terms of the diamagnetic coefficients and the linewidth broadening, where the magneto-PL spectra of single laterally coupled QD structures of excitons (X_1_ and X_2_), local biexcitons (X_1_X_1_ and X_2_X_2_), and a coupled biexciton (X_1_X_2_) were measured for *θ*_ext_ = 90° excitation. While the two excitons of a local biexciton are isolated in the same QD, the different excitons of a coupled biexciton are bound across separate QDs. Therefore, the PL spectrum of a coupled biexciton (X_1_X_2_–X_2_) shows a significantly larger diamagnetic coefficient (68 μeVT^−2^) than those of local biexcitons and excitons (37–42 μeVT^−2^) due to the larger wavefunction extent^27^. Although the X_1_X_2_–X_2_ transition was not clearly separated from the X_2_ PL spectrum, the overlapping linewidth of X_1_X_2_–X_2_ and X_2_ broadened significantly up to ~380 μeV from ~280 μeV with increasing external magnetic field up to 8 T. The linewidth of the other PL spectra remained small (290 μeV) at 8 T. Therefore, we infer that the X_1_X_2_–X_2_ transition spectrally overlaps with the X_2_ spectrum, but the transition rate is small compared with that of X_1_X_2_-X_1._ As far as the cascade photon pair emission between the coupled biexcitons and excitons is concerned, we also found that the polarization pair is perpendicular to each other. For example, the polarized transition of X_1_X_2_–X_1_ along *θ*_ext_ = 0° likely pairs with the polarized transition of X_1_–G along *θ*_ext_ = 90°.

In Fig. 2c, the PL anisotropies of excitons, local biexcitons, and a coupled biexciton are shown in terms of the normalized intensity as a function of *θ*_det_, and all show the same anisotropy with a degree of polarization of 74%. On the other hand, with excitation polarized perpendicular to the coupled direction (*θ*_ext_ = 0°) (Fig. 2d), the polarized PL spectrum at the same orientation (*θ*_det_ = 0°) is enhanced (Fig. 2e) compared with that with *θ*_ext_ = 90° excitation (Fig. 2b). It is noticeable that the polarized PL intensity at *θ*_det_ = 90° still dominates despite the perpendicularly polarized excitation (*θ*_ext_ = 0°). The polarized PL along the coupled direction (*θ*_det_ = 90°) is always dominant regardless of the excitation polarization due to the structural anisotropy. However, the polarization anisotropy can be controlled by changing the direction of the excitation polarization. As shown in Fig. 2f, the PL anisotropy with *θ*_ext_ = 0° excitation is suppressed, with a decreased degree of polarization (10%) compared with that with *θ*_ext_ = 90° excitation (74%). The binding energy of the two local biexcitons (X_1_X_1_ and X_2_X_2_) generated by *θ*_ext_ = 0° excitation increases to ~5 meV, but a coupled biexciton (X_1_X_2_) is not generated by *θ*_ext_ = 0° excitation. Therefore, both the wavefunction extent of local biexcitons and the interaction between two separate excitons in laterally coupled QDs are affected by *θ*_ext_. As shown schematically in Fig. 2d, the wavefunction extents is enhanced via *E*_loc_ for the excitation polarization of *θ*_ext_ = 0°, but this alignment orientation suppresses the binding of X_1_ and X_2_. To evaluate the elliptical shape of the wavefunctions^38^, we also determined the eccentricity $e\,=\,\sqrt {1\,-\,\left( {{\it{I}}\left( {0^o} \right)\!/\!\left( {{\it{I}}\left( {90^o} \right)} \right)^2} \right.^{}}$ from the anisotropic PL intensity for *θ*_det_, where *I*(0°) and *I*(90°) are the normalized PL intensities at *θ*_det_ = 0° and *θ*_det_ = 90°, respectively. With polarized excitation at *θ*_ext_ = 90° (Fig. 2c), the anisotropic normalized PL intensity for *θ*_det_ gives *e* = 0.99. On the other hand, when the excitation polarization angle is rotated to *θ*_ext_ = 0° (Fig. 2f), the eccentricity decreases to 0.57. In addition, the PL spectrum energies of excitons and local biexcitons increase with *θ*_ext_ = 0° excitation (Fig. 2e) compared with those with *θ*_ext_ = 90° (Fig. 2b). As shown schematically in Fig. 2d, *θ*_ext_ = 0° excitation seems to cause suppression of the wavefunction extent along the coupled direction via *E*_loc_. As a result, the confinement energy is increased, and the shape ellipticity decreases. On the other hand, the wavefunction extent perpendicular to the coupled direction is relatively increased, giving rise to a PL enhancement along *θ*_det_ = 0°.

**Fig. 3:**
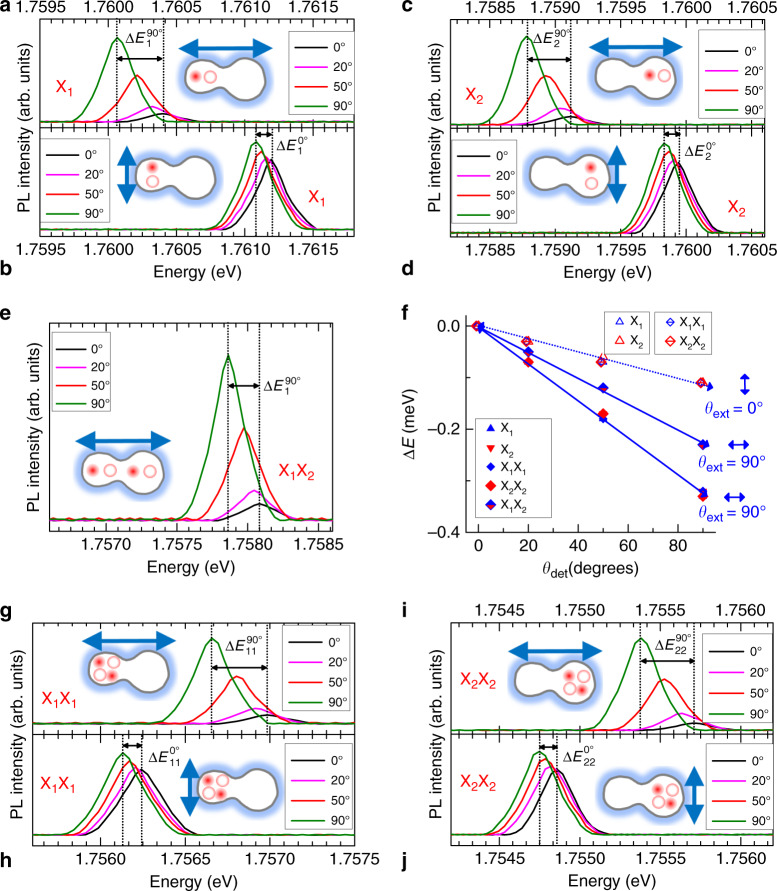
Schematic of the excitation polarization dependence of the PL emission. With polarized excitation (*θ*_ext_ = 90° or *θ*_ext_ = 0°), the along *θ*_det_ dependences of the polarized PL spectra at X_1_ (**a**, **b**) and X_2_ (**c**, **d**) are compared. **e***θ*_det_ dependence of the polarized PL spectrum at X_1_X_2_ under *θ*_ext_ = 90° excitation. For the two perpendicularly polarized excitations of *θ*_ext_ = 90° and *θ*_ext_ = 0°, the polarized PL peak energies of excitons and biexcitons are plotted for various *θ*_det_ (**f**), and the polarized PL spectra at X_1_X_1_ (**g**, **h**) and X_2_X_2_ (**i**, **j**) are also shown for various *θ*_det_

With a fixed excitation of either *θ*_ext_ = 90° or *θ*_ext_ = 0°, we found that the PL spectra of the laterally coupled QD structures show an energy difference (Δ*E*) with increasing *θ*_det_ from 0° to 90°. To represent the redshift, the energy difference ΔE was defined with negative values as shown in Fig. 3f. For example, Δ*E*_1_^90^ represents the redshift of X_1_ under *θ*_ext_ = 90° excitation. With excitation polarized along the coupled direction (*θ*_ext_ = 90°), both X_1_ (Fig. 3a) and X_2_ (Fig. 3c) show the same redshift (−0.33 meV) in the perpendicularly polarized PL spectra at *θ*_det_ = 0° and *θ*_det_ = 90° despite the size difference of the two QDs. On the other hand, with excitation polarized perpendicular to the coupled direction (*θ*_ext_ = 0°), a decreased redshift of −0.11 meV is obtained for both X_1_ (Fig. 3b) and X_2_ (Fig. 3d) from the two perpendicularly polarized PL spectra. If we assume that the polarized PL intensity is associated with the exciton oscillator strength at *θ*_det_, then the anisotropy of the PL intensity may characterize the shape of the exciton wavefunction. Therefore, the lateral wavefunctions of X_1_ and X_2_ are elongated along the coupled direction, as shown schematically in Fig. 2a. The lateral anisotropy of the wavefunction extent may also explain the gradual redshift with increasing *θ*_det_ from 0° to 90°. Interestingly, X_1_X_2_ shows an energy difference of 0.22 meV for the two perpendicular polarized PL spectra at *θ*_det_ = 0° and *θ*_det_ = 90° (Fig. 3e).

In Fig. 3f, the *θ*_det_ dependences of the redshift (Δ*E*) are plotted for the two perpendicular excitations of *θ*_ext_ = 90° and *θ*_ext_ = 0°. Given polarized excitation at either *θ*_ext_ = 90° or *θ*_ext_ = 0°, both excitons (X_1_ and X_2_) and local biexcitons (X_1_X_1_ and X_2_X_2_) of QD_1_ and QD_2_ give rise to the same Δ*E* for *θ*_det_. As shown in Fig. 3g, i, the polarized excitation of *θ*_ext_ = 90° gives rise to the same energy difference (3*Δ* = 0.33 meV) in the perpendicularly polarized PL spectra of both local biexcitons and excitons. On the other hand, with the polarized excitation of *θ*_ext_ = 0°, the same decreased energy difference (*Δ* = 0.11 meV) is obtained in the perpendicularly polarized PL spectra of both local biexcitons and excitons (Fig. 3h, j). These results support the assertion that the orientation of the polarized excitation affects the wavefunction anisotropy of both excitons and biexcitons in laterally coupled QD structures.

**Fig. 4:**
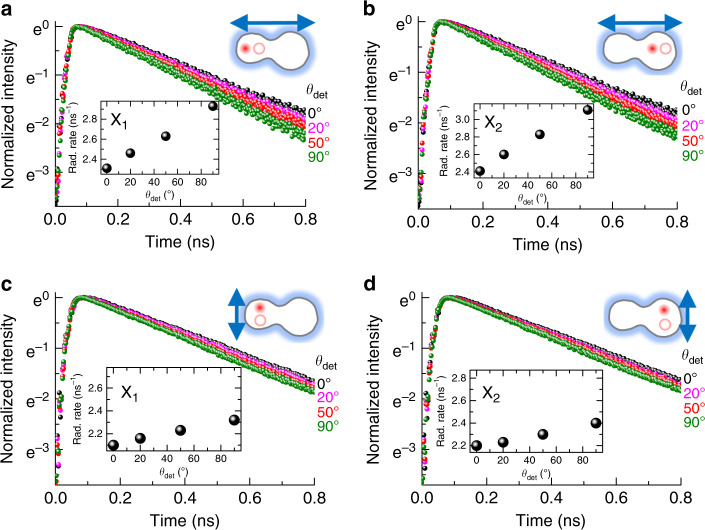
Schematic of the polarization dependence of the time-resolved emission. With two polarized excitations of *θ*_ext_ = 90° and *θ*_ext_ = 0°, the time-resolved PL of X_1_ (**a**, **c**) and X_2_ (**b**, **d**) is observed for changing detection angle θ_det_, where each inset shows the PL decay rate for *θ*_det_

When considering the dot size (a few tens of nanometers) of laterally coupled QDs, the oscillator strength of excitons is expected to increase with increased confinement size^40^. Therefore, the anisotropic wavefunction extent in a laterally coupled QD may give rise to an anisotropic radiative decay rate. With the two perpendicularly polarized excitations of *θ*_ext_ = 90° and *θ*_ext_ = 0°, we measured the time-resolved PL of X_1_ (Fig. 4a, c) and X_2_ (Fig. 4b, d) at various *θ*_det_. As shown in the insets, the PL decay rates of the excitons were also plotted as *θ*_det_ increases from *θ*_det_ = 0° to *θ*_det_ = 90°. When the excitation is polarized at *θ*_ext_ = 90° (Fig. 4a, b), the PL decay rates of X_1_ and X_2_ increase significantly with increasing *θ*_det_ up to *θ*_det_ = 90°. On the other hand, with polarized excitation at *θ*_ext_ = 0° (Fig. 4c, d), the difference in the exciton PL decay rates at *θ*_det_ = 0° and *θ*_det_ = 90° is relatively small. Because non-radiative decay is unavoidable in PL, the observed PL decay rate is different from the corresponding radiative decay rate. Nevertheless, the anisotropic PL decay rate is likely associated with the anisotropic wavefunction. X_2_ shows a larger PL decay rate (3.1 ns^−1^) than X_1_ (2.9 ns^−1^) at *θ*_det_ = 90° when the excitation is polarized at *θ*_ext_ = 90°. With the polarization excitation of *θ*_ext_ = 0°, X_2_ still shows a larger PL decay rate (2.4 ns^−1^) than X_1_ (2.3 ns^−1^) at *θ*_det_ = 90°. These results are consistent with the confinement size dependence of the exciton oscillator strength; i.e., the larger the wavefunction extent at *θ*_det_ is, the higher the radiative decay rate. Because the size of QD_2_ is slightly larger than that of QD_1_, a relatively large oscillator strength is expected in QD_2_ compared with that in QD_1_ at a given orientation (*θ*_det_).

**Fig. 5:**
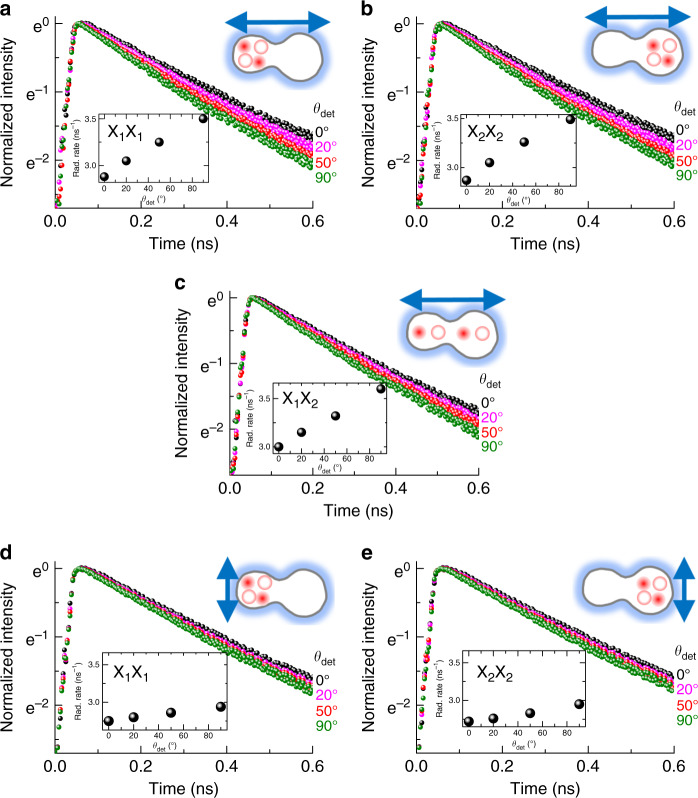
Schematic of the time-resolved emission from local and coupled biexcitons. With polarized excitation at *θ*_ext_ = 90°, the time-resolved PL of two local biexcitons (X_1_X_1_ and X_2_X_2_) (**a**, **c**) and coupled biexcitons (X_1_X_2_) (**b**) is plotted as a function of the detection angle *θ*_ext_. With polarized excitation at *θ*_ext_ = 0°, the *θ*_det_ dependence of the time-resolved PL is also shown for the two local biexcitons (X_1_X_1_ and X_2_X_2_) (**d**, **e**). All insets show the PL decay rate as *θ*_det_ varies

The *θ*_det_ dependence of the time-resolved PL was also measured for local biexcitons (Fig. 5a, c) and coupled biexcitons (Fig. 5d, e) with the two polarized excitations of *θ*_ext_ = 90° and *θ*_ext_ = 0°. The PL decay time for all the biexcitons is significantly shorter than that for the excitons, but the *θ*_det_ dependence of the monotonic PL decay rate is similar to that for excitons. As *θ*_det_ increases from 0° to 90°, the PL decay rate of all biexcitons increases and is maximized at *θ*_det_ = 90°. With polarized excitation at *θ*_ext_ = 90°, we found that the PL decay rate for local biexcitons (X_1_X_1_ and X_2_X_2_) measured at a given *θ*_det_ is significantly larger than that obtained at the same *θ*_det_ with polarized excitation at *θ*_ext_ = 0°. Therefore, we conclude that the wavefunction anisotropy of biexcitons is also shaped by changing *θ*_ext_, resulting in an anisotropic PL decay rate. Interestingly, the PL decay rate of coupled biexcitons (X_1_X_2_) is larger than that of local biexcitons (X_1_X_1_ and X_2_X_2_). Because the wavefunction extent of coupled biexcitons is larger than that of local biexcitons, the coupled biexcitons are expected to have a relatively large oscillator strength. Hence, it is reasonable that the radiative decay rate of coupled biexcitons is larger than that of local biexcitons, and this result is also consistent with the large diamagnetic coefficient of coupled biexcitons compared with that of local biexcitons.

In conclusion, we found that a laterally coupled QD structure shows anisotropy in the PL intensity, where the polarized PL intensity at *θ*_det_ = 90° always dominates in laterally coupled QDs, and the degree of polarization varies from 10 to 74% with changing orientation of the excitation polarization from *θ*_ext_ = 0° to *θ*_ext_ = 90°. We found that both excitons in two separate QDs show the same anisotropy in their time-integrated PL intensity and PL decay rate, and this result can be attributed to a wavefunction anisotropy of excitons and biexcitons, where the local electric field is possibly mediated and the dipole–dipole interaction plays an important role. Although the polarized light excites carriers in the barrier, the PL anisotropy in the ground states is strongly affected by the orientation of the polarized excitation via *E*_loc_. Therefore, the anisotropic wavefunction can be shaped by the polarization of nonresonant excitation.

## Materials and methods

Laterally coupled QDs were grown by a VG80 solid-source molecular beam epitaxy system on a GaAs (001) substrate. A 200-nm-thick GaAs buffer layer and a 100-nm-thick Al_0.3_Ga_0.7_As barrier were grown at the substrate temperature (*T*_s_) of 580 °C. For the formation of Ga droplets, *T*_s_ was decreased to 300 °C. The total amount of Ga deposited on the Al_0.3_Ga_0.7_As surface comprised five monolayers, with a deposition rate of 0.1415 nm/s without arsenic supply. For the fabrication of laterally coupled GaAs QDs, As_4_ with a beam equivalent pressure of 1.5 × 10^−5^ Torr was supplied. The anisotropy of Ga diffusion may be the major driving force for the formation of laterally coupled QDs along the [1$\bar 1$0] direction on the (001) surface. After the formation of Ga droplets, *T*_s_ was subsequently decreased to 200 °C to suppress Ga diffusion during the subsequent As supply. The PL of laterally coupled QDs was collected at 4 K using a confocal arrangement with a spot size of 0.8 μm^2^. For time-resolved PL measurements, a time-correlated single photon counting system was used, excited by a frequency-doubled (400 nm) Ti:sapphire laser with a 120 fs pulse duration at an 80-MHz repetition rate (the detailed setup can be found in the Supplementary Information).

## Supplementary information

Supplementary

## References

[1] Bayer M (2001). Coupling and entangling of quantum states in quantum dot molecules. Science.

[2] Petta JR (2005). Coherent manipulation of coupled electron spins in semiconductor quantum dots. Science.

[3] Bester G, Shumway J, Zunger A (2004). Theory of excitonic spectra and entanglement engineering in dot molecules. Phys. Rev. Lett..

[4] Robledo L (2008). Conditional dynamics of interacting quantum dots. Science.

[5] Sheng WD, Leburton JP (2002). Anomalous quantum-confined stark effects in stacked InAs/GaAs self-assembled quantum dots. Phys. Rev. Lett..

[6] Emary C, Sham LJ (2007). Optically controlled logic gates for two spin qubits in vertically coupled quantum dots. Phys. Rev. B.

[7] Weiss KM (2012). Coherent two-electron spin qubits in an optically active pair of coupled InGaAs quantum dots. Phys. Rev. Lett..

[8] Villas-Bôas JM, Govorov AO, Ulloa SE (2004). Coherent control of tunneling in a quantum dot molecule. Phys. Rev. B.

[9] Xu XL, Williams DA, Cleaver JAR (2005). Splitting of excitons and biexcitons in coupled InAs quantum dot molecules. Appl. Phys. Lett..

[10] Vora PM (2015). Spin-cavity interactions between a quantum dot molecule and a photonic crystal cavity. Nat. Commun..

[11] Kerfoot ML (2014). Optophononics with coupled quantum dots. Nat. Commun..

[12] Thierschmann H (2015). Three-terminal energy harvester with coupled quantum dots. Nat. Nanotechnol..

[13] Rontani M (2004). Molecular phases in coupled quantum dots. Phys. Rev. B.

[14] Zhou XR (2013). Coulomb interaction signatures in self-assembled lateral quantum dot molecules. Phys. Rev. B.

[15] Zhou XR, Doty M (2014). Design of 4-electrode optical device for application of vector electric fields to self-assembled quantum dot complexes. J. Appl. Phys..

[16] Doty MF (2009). Antibonding ground states in InAs quantum-dot molecules. Phys. Rev. Lett..

[17] Ma XY (2016). Hole spins in an InAs/GaAs quantum dot molecule subject to lateral electric fields. Phys. Rev. B.

[18] De La Giroday AB (2011). Excitonic couplings and Stark effect in individual quantum dot molecules. J. Appl. Phys..

[19] Ortner G (2005). Control of vertically coupled InGaAs/GaAs quantum dots with electric fields. Phys. Rev. Lett..

[20] Kagan CR, Murray CB (2015). Charge transport in strongly coupled quantum dot solids. Nat. Nanotechnol..

[21] Wijesundara KC (2011). Tunable exciton relaxation in vertically coupled semiconductor InAs quantum dots. Phys. Rev. B.

[22] Stinaff EA (2006). Optical signatures of coupled quantum dots. Science.

[23] Krenner HJ (2006). Optically probing spin and charge interactions in a tunable artificial molecule. Phys. Rev. Lett..

[24] Wang LJ (2009). Self-assembled quantum dot molecules. Adv. Mater..

[25] Liang BL (2008). Energy transfer within ultralow density twin InAs quantum dots grown by droplet epitaxy. ACS Nano.

[26] Unold T (2005). Optical control of excitons in a pair of quantum dots coupled by the dipole–dipole interaction. Phys. Rev. Lett..

[27] Kim H (2016). Exciton dipole–dipole interaction in a single coupled-quantum-dot structure via polarized excitation. Nano Lett..

[28] Beyer J (2009). Spin injection in lateral InAs quantum dot structures by optical orientation spectroscopy. Nanotechnology.

[29] Cundiff ST (1996). Optical coherence in semiconductors: strong emission mediated by nondegenerate interactions. Phys. Rev. Lett..

[30] Guenther T (2002). Coherent nonlinear optical response of single quantum dots studied by ultrafast near-field spectroscopy. Phys. Rev. Lett..

[31] Kim H (2018). Light controlled optical Aharonov–Bohm oscillations in a single quantum ring. Nano Lett..

[32] Santori C (2004). Submicrosecond correlations in photoluminescence from InAs quantum dots. Phys. Rev. B.

[33] Sallen G (2010). Subnanosecond spectral diffusion measurement using photon correlation. Nat. Photonics.

[34] Wang ZM (2008). Unusual role of the substrate in droplet-induced GaAs/AlGaAs quantum-dot pairs. Phys. Status Solidi Rapid Res. Lett..

[35] Keizer JG (2010). Atomic scale analysis of self assembled GaAs/AlGaAs quantum dots grown by droplet epitaxy. Appl. Phys. Lett..

[36] Takagahara T (2000). Theory of exciton doublet structures and polarization relaxation in single quantum dots. Phys. Rev. B.

[37] Hafenbrak R (2007). Triggered polarization-entangled photon pairs from a single quantum dot up to 30 K. N. J. Phys..

[38] Kim HD (2013). Asymmetry of localised states in a single quantum ring: polarization dependence of excitons and biexcitons. Appl. Phys. Lett..

[39] Kodriano Y (2010). Radiative cascade from quantum dot metastable spin-blockaded biexciton. Phys. Rev. B.

[40] Hours J (2005). Exciton radiative lifetime controlled by the lateral confinement energy in a single quantum dot. Phys. Rev. B.

[41] Adachi S (2003). Exciton-exciton interaction and heterobiexcitons in GaN. Phys. Rev. B.

