# Supplementary material for: Optical shaping of the polarization anisotropy in a laterally coupled quantum dot dimer
Source: Light Sci Appl. 2020 Jun 11;9:100. doi: 10.1038/s41377-020-0339-3 (PMC7286917; doi:10.1038/s41377-020-0339-3)
Supplement: Supplementary file 1 — Supplementary [file 41377_2020_339_MOESM1_ESM.doc]

**Supplementary Information for**

**Optical shaping of the polarization anisotropy in a laterally coupled quantum dot dimer**

**Heedae Kim^1,2^, Kwangseuk Kyhm^3,*^, Robert A. Taylor^2,**^, Jong Su Kim^4^, Jin Dong Song^5^, and Sungkyun Park^6^**

^1^School of Physics, Northeast Normal University, Changchun 130024, China

^2^Clarendon Laboratory, Department of Physics, University of Oxford, Oxford, OX1 3PU, U.K.

^3^Department of Opto-mechatronics, Pusan Nat’l University, Busan 609-735, Republic of Korea

^4^Department of Physics, Yeungnam University, Gyeongsan, 712-749, Republic of Korea

^5^Nano-Photonics Research Center, KIST, Seoul, 136-791, Republic of Korea

^6^Department of Physics, Pusan Nat’l University, Busan 609-735, Republic of Korea

* kskyhm@pusan.ac.kr

** robert.taylor@physics.ox.ac.uk

**Sample growth**

Fig. S1 shows a schematic illustration of the formation of various GaAs quantum structures as the amount of As flux is varied. The upper row of Fig. S1 shows the cross-sectional shape for the crystallization process in conceptual form (Ga droplets change into GaAs quantum structures during crystallization under the influence of the As supply). The crystallization process can be explained by the variation of the V/III ratio, which is the driving force for the GaAs crystallization. When the V/III ratio is relatively high (As flux ~ 10^-4^ Torr), all the areas of the Ga droplets can be effectively crystallized. Also, the relatively large amount of As atoms may block the out-migration of Ga atoms from the Ga droplets. As a result, GaAs QDs can form as shown in Fig. S1 (high As flux) and (d). When a medium As flux (~ 10^-5^ Torr) is supplied, anisotropic Ga migration occurs along to the [1**
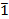
**0] direction because the As dimer now is parallel to the [1**
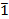
**0] direction with a (2 × 4) surface reconstruction. Similarly, a preferential out-migration of the Ga atoms along the [1**
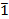
**0] direction from the Ga droplets may be seen when compared with the [110] direction. As a result, the shape of the GaAs nanostructure can be asymmetric (J. S. Kim et al., Journal of the Korean Physical Society 73, 190 (2018)). With a further decrease in the As flux, the shape of the asymmetric GaAs nanostructure becomes symmetric once more, as shown in Fig. S1(f). When the V/III ratio is low (As flux × 10^-6^ Torr), the crystallization process may be slower than that for the medium V/III ratio, because the incorporation rate between Ga and As is small. This slow process results in an increase in the out-migration probability of the Ga atoms from the droplet, as schematically described in Fig. S1 (low As flux) and (f), resulting in formation of GaAs quantum rings with a symmetric shape. At low As flux, the migration length of the Ga atoms along the [1**
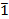
**0] direction reaches a limit, the out-migration of the Ga atoms toward the [110] direction can be significantly increased, resulting in the formation of the relatively symmetric QRs. In previous works, we confirmed that the out-migration properties of the Ga atoms are strongly dependent on the crystal direction and the surface reconstructions caused by the As flux (A. Ohtake et al., Phys. Rev. Lett. 89, 206102 (2002), I. T. Ito et al., Appl. Surf. Sci. 237, 194 (2004)).


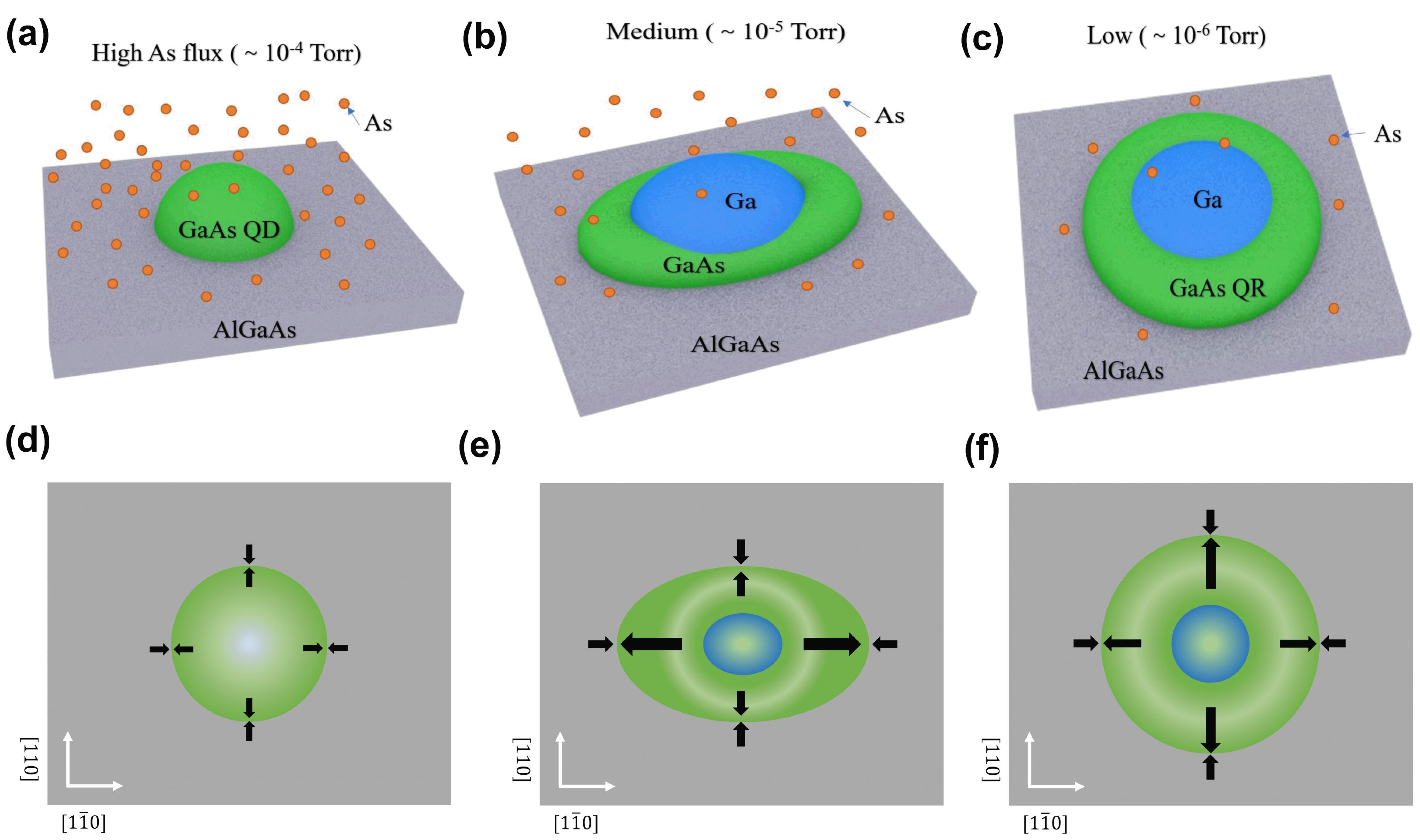


**Fig. S1:** Various GaAs quantum structures are formed by changing the amount of the As flux.

To fabricate the laterally-coupled GaAs QDd (CQD), the sample was grown by a VG80 solid-source molecular beam epitaxy (MBE) system on a GaAs (001) substrate. A 200 nm thick GaAs buffer layer and a 100 nm thick Al_0.3_Ga_0.7_As barrier were grown at the substrate temperature (T_s_) of 580^o^C. For the formation of Ga droplets, T_s_ was decreased to 300^o^C. The details of T_s_ calibration and chamber conditions for the DE were discussed in (J. S. Kim et al., Appl. Phys. Lett. 88, 241911 (2006)) and (J. S. Kim et al., Appl. Phys. Lett. 85, 5893 (2004)). The total amounts of Ga deposited on the Al_0.3_Ga_0.7_As surface were 5 monolayers (ML) with a deposition rate of 0.1415nm/s without arsenic supply. After the formation of Ga droplets, T_s_ was subsequently decreased to 200^o^C to suppress the Ga diffusion during the subsequent As supply. For the fabrication of laterally-coupled GaAs CQD by using the V/III ratio effect, As_4_ with a beam equivalent pressure of 1.5 × 10^-5^ Torr was supplied. The anisotropy of the Ga out-migration may be the major driving force for the formation of laterally-coupled GaAs CQD along the direction of [1**
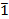
**0] on the (001) surface.


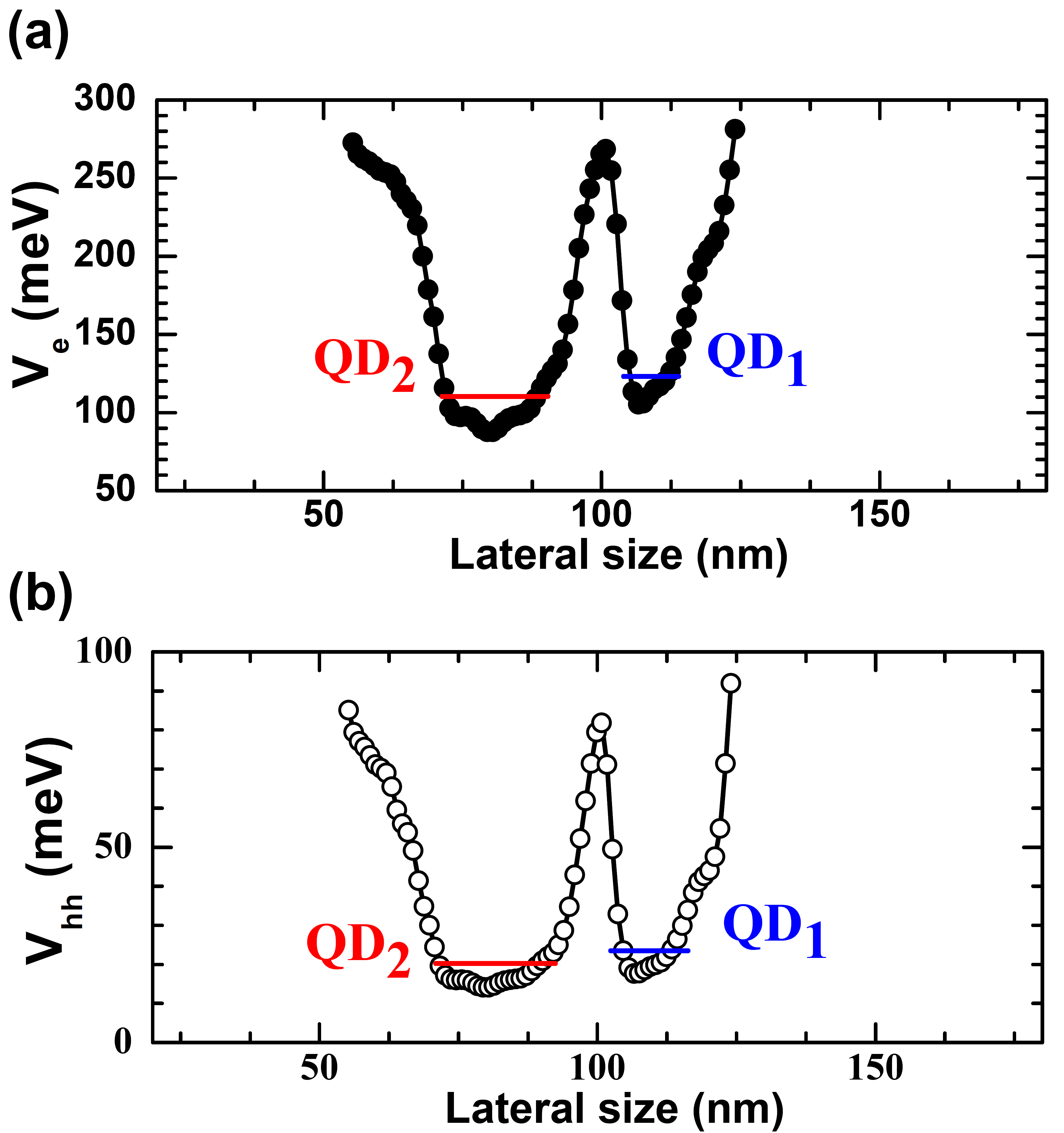


**Fig. S2:** The confinement potential for electrons (a) and holes (b) calculated from Fig. 1(a).

**Confinement potential valleys of laterally-coupled CQDs**

Given the AFM image from Fig.1(a), we calculated the confinement potential valleys of a laterally-coupled CQD structure for electrons (Fig. S2(a)) and holes (Fig. S2(b)), respectively. By using the parabolic approximation, the ground states of an independent electron and hole in the conduction and valence bands were also obtained for the two QDs, respectively. In order to estimate the exact exciton confinement energy, the whole morphology is required. Nevertheless, Fig. 1(a) provides enough information to estimate the potential separation (~12 nm). Therefore, a wavefunction overlap via tunnelling is very inefficient, and the two excitons of separate QDs interact through the dipole-dipole interaction. In this case, the alignment orientation of the two dipoles is crucial if a bonding state is to be produced.

**Experimental setup of micro-PL**

The micro-photoluminescence (PL) setup is shown in Fig. S3. For light excitation a Ti:sapphire laser was used with 120 fs pulse duration at an 80 MHz repetition rate. The fundamental 800 nm pulses were frequency doubled by a BBO crystal for excitation at 400 nm. The residual 800 nm was detected by a fast photo-diode for triggering a time-correlated single photon counting system (TCSPC). In order to measure the PL from a single CQD structure, a pinhole and an objective lens were used, and the spatial image was taken by a camera to find the focal optimum. The excitation spot size was 0.8 μm^2^ and sharp PL spectra from a single CQDs was measured by a charge coupled device (CCD) camera attached to a monochromator with a spectral resolution of 0.75 nm, and a photomultiplier tube (PMT) was used as the detector for the time-resolved PL measurement. The sample was mounted in a cryostat and all measurements were performed at 4 K.


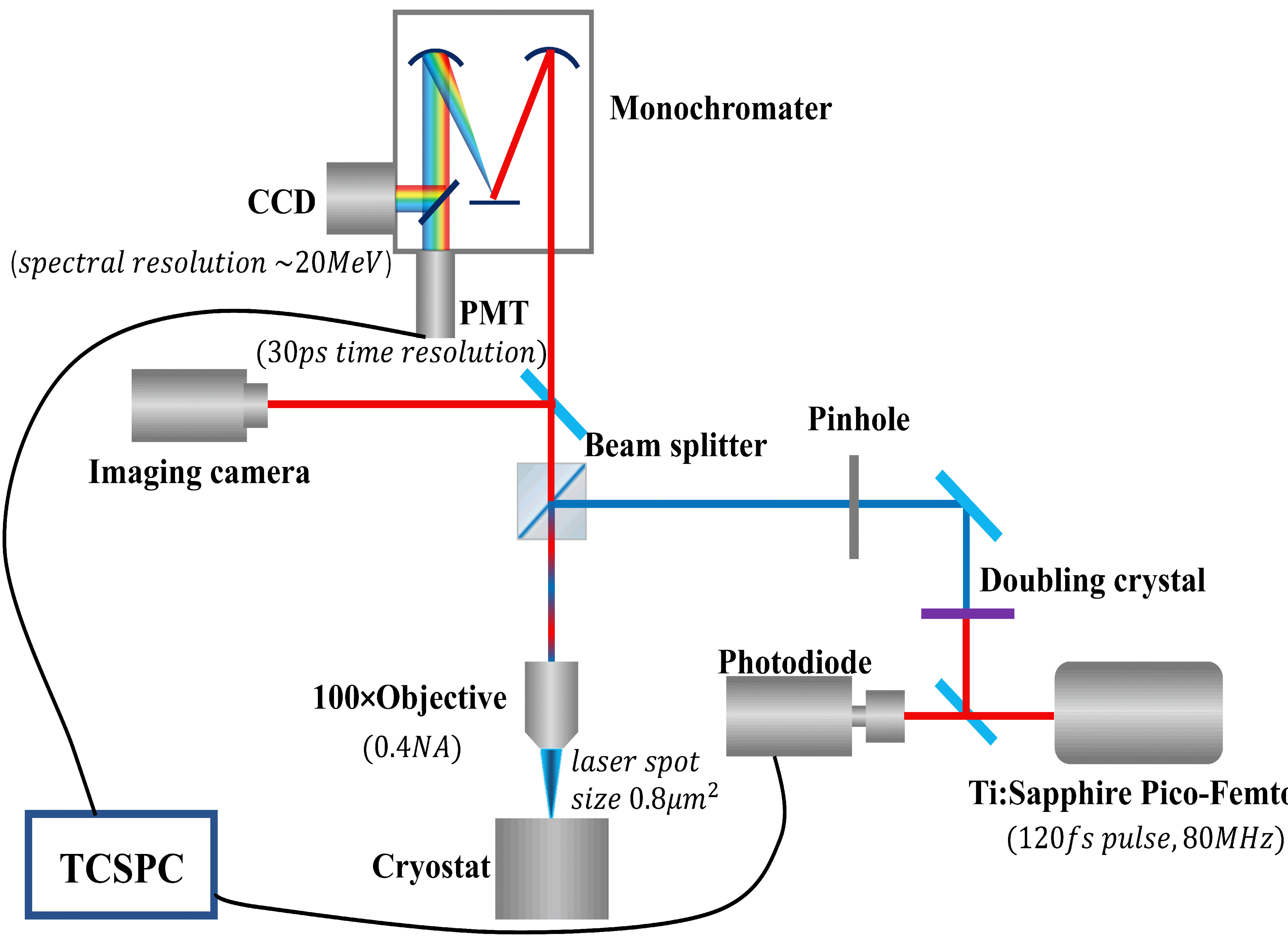


**Fig. S3:** Micro-photoluminescence setup for a single laterally-coupled CQD.
